# Radio(chemo)therapy with curative intent for anal cancer – effectiveness and toxicity in elderly vs. younger patients

**DOI:** 10.3389/fonc.2025.1567655

**Published:** 2025-05-16

**Authors:** Mahalia Zoe Anczykowski, Polina Rösel, David Alexander Ziegler, Laura Anna Fischer, Manuel Guhlich, Rami A. El Shafie, Stefan Rieken, Leif Hendrik Dröge, Martin Leu

**Affiliations:** ^1^ Department of Radiotherapy and Radiation Oncology, University Medical Center Göttingen, Göttingen, Germany; ^2^ Göttingen Comprehensive Cancer Center (G-CCC), University Medical Center Göttingen, Göttingen, Germany; ^3^ Department of Radiation Oncology and Radiotherapy, Medical University Lausitz – Carl Thiem, Cottbus, Germany

**Keywords:** anal cancer, primary treatment, radiotherapy, elderly patients, geriatric oncology, comorbidities, toxicity, survival

## Abstract

**Background:**

Primary radio(chemo)therapy is a therapeutic standard strategy for advanced anal squamous cell carcinoma (ASCC). For elderly patients evidence concerning long-term oncological outcome is scarce.

**Methods:**

160 patients with advanced ASCC treated primarily by radio(chemo)therapy with curative intent were included. Baseline characteristics such as the Charlson Comorbidity Index as well as treatment-associated and long-term oncologic outcomes of patients with advanced (≥ 70 years) and younger (< 70 years) age were compared.

**Results:**

Elderly patients had more comorbidities. They less frequently received concomitant chemotherapy. Acute enteritis ≥ III° and late pelvic bone fracture occurred more frequently in elderly patients. Overall survival and progression-free survival estimates were significantly lower for elderly patients, respectively (OS: HR 2.53, 95% CI 1.54-4.18; p < 0.001 and PFS: HR 2.10, 95% CI 1.29-3.42; *p* = 0.003). Locoregional and distant control did not show significant differences between elderly vs. younger patients.

**Conclusion:**

Primary radio(chemo)therapy seems to be an effective and relatively safe treatment option also in elderly patients. The lower overall and progression-free survival estimates as well as the negative survival influence of a higher comorbidity index strengthen the necessity to comprehensively weighing up and discuss potential benefits and side effects of primary radio(chemo)therapy.

## 1 Introduction

Anal carcinomas are relatively rare malignancies (1–3). The cumulative estimated incidence was 54–194 new cases worldwide in 2022 (3). An increasing incidence was reported for several countries such as for example the US, Canada, the UK and Germany (1).

The recommended treatment for advanced anal carcinomas without distant metastasis is radiochemotherapy (4). Although especially older adults have an increased risk of anal cancer (5), studies that have been instrumental in establishing this therapeutic standard predominantly included relatively young and/or fit patients. Comorbid and/or older patients were underrepresented or even excluded (2, 6–12). A paucity of retrospective studies investigated the treatment of elderly patients. To the best of our knowledge only four of them report long-term oncological outcome data (13–16).

The present study aims to contribute to a deeper characterization and more individualized, needs-adapted treatment of elderly patients with the rare disease of anal carcinoma. Therefore, it was investigated whether elderly ASCC patients routinely receive R(C)T with curative intent and how feasible and safe the treatment is for them. Potential factors with impact on prognosis and therefore potential objective criteria to guide treatment selection were analyzed.

## 2 Patients and methods

### 2.1 Patients

A retrospective analysis of patients with non-metastatic ASCC treated by radio(chemo)therapy (R(C)T) with curative intent was performed. The study was conducted at an academic tertiary referral center. Time span of inclusion was 03/1992 to 11/21. Patients were identified and data were obtained from the hospital's cancer database and the original medical records. The study was authorized by the institutional review board ("Ethikkommission der Universitätsmedizin Göttingen"; 7/1/21 and 41/3/21) and conducted in accordance with the national regulation and the Helsinki Declaration. Data of some patients were partially included in previous studies (17, 18). "Elderly patients" were defined as persons aged 70 years or older at the time of initial diagnosis. Patients with prior pelvic RT were excluded from this study.

### 2.2 Staging and treatment

Staging examinations were performed as described previously (18). Diagnosis was confirmed histopathologically. Staging was performed in accordance with the eighth edition of the Union for International Cancer Control's (UICC) "TNM Classification of Malignant Tumours" (19).

Patient-specific oncological treatment concepts were developed on an interdisciplinary basis in accordance with the current guidelines (20–22). Primary R(C)T was standardly performed in patients with advanced anal cancer. In certain instances, surgical intervention was initially used for cases exhibiting initially localized findings, which were deemed resectable through surgical means alone. However, the decision was subsequently taken to utilize primary R(C)T due to the presence of more extensive findings. Detailed radiotherapeutic treatment procedures were previously described (17, 18). In brief, for treatment planning, patients underwent planning CT scans of the pelvic region. The target volumes included the primary tumor region and mesorectal, iliac, and inguinal lymph nodes. A total dose of 50.4 Gy in 1.8 Gy fractions five times a week constituted the standard radiotherapeutic treatment regime. Different total doses may have been prescribed by the treating radiation oncologist in individual cases with e.g. small primary or very advanced tumors. Treatment was standardly performed in prone position. Patients were instructed to present with a comfortably and always as equal as possible filled bladder and empty bowel. To prevent excessive intestinal gas formation dietary recommendations were given. RT delivery techniques used were conventional 2D-/3D-RT with individualized treatment fields or dynamic- RT (Intensity-modulated radiation therapy (IMRT) or Volumetric modulated arc therapy (VMAT)). IMRT and VMAT plans were generated using Eclipse (Varian Medical Systems, Palo Alto, CA, USA). Organ at risk (OAR) constraints were defined based on the QUANTEC recommendations (23, 24). Concerning 3D-RT dose exposure to OARs was evaluated individually and left at the treating physician's discretion.

### 2.3 Concomitant chemotherapy

Concomitant chemotherapy (CTx) is part of the standard curative treatment concept for (advanced) anal cancer. The chemotherapeutic treatment procedures were already described previously (17, 18). In brief, prior to treatment initiation a comprehensive clinical assessment of the patient's individual state of health was performed. In addition to the staging examinations, capability to tolerate CTx was evaluated. The additional examinations included at least laboratory analyses with blood cell count and clinical chemistry, an electrocardiogram as well as pulmonary function analysis. Dihydropyrimidine dehydrogenase testing prior to the application of 5-fluorouracil (5-FU) has also been part of the clinical standard at the latest since 2014. A certain regimen was recommended by the treating physician on an individual basis taking into account the present evidence and the potential benefits and risks. The first-choice standard was usually a CTx regimen consisting of 5-fluorouracil (d1–4, d29–32, 1000 mg/qm of body surface area/d) and mitomycin c (d1, d29, 10 mg/qm of up to a body surface area of two qm) (17, 18).

### 2.4 Toxicity scoring and follow-up procedures

During R(C)T clinical visits and laboratory analyses with toxicity assessments were performed at least weekly. Toxicities were scored in accordance with the Common Terminology Criteria for Adverse Events (CTCAE) Version 5.0 (25) and Late Effects of Normal Tissues/Subjective Objective Management Analysis (LENT/SOMA) criteria (26). After completion of R(C)T, the care concept included at least five years of follow-up. Patients were assessed on a standardized basis at 18-month intervals up to 54 months during the follow-up period in the radiotherapeutic clinic. Furthermore, a more frequent follow-up was conducted by the responsible gastroenterologist or surgeon.

### 2.5 Endpoints

Time dependent endpoints included overall survival (OS), progression-free survival (PFS), locoregional control (LRC), distant control (DC) and, among patients without pre-therapeutic colostomy, stoma-free survival (SFS). Date of the histopathological diagnosis was considered as starting point for the analyses. Concerning OS, death from any cause was counted as event. PFS was defined as time until locoregional and/or distant tumor progression or death from any cause. Regarding LRC, local and regional recurrences were considered as event. For DC, the occurrence of distant metastasis was counted as event. SFS was defined as time until colostomy or death from any cause.

### 2.6 Statistics

Descriptive analysis of values or quantities included the calculation of the respective mean value and corresponding standard deviation, median and/or absolute, and relative frequencies. Chi-square test or Kruskal-Wallis test were used to analyze frequency distributions. The Kaplan-Meier method was applied to calculate the above-mentioned time-dependent endpoints (27). Log-rank test was used to compare survival times.

Cox regression proportional hazard models were calculated to analyze the influence of variables on survival. Variables that were found to be significant in the univariable analysis were subsequently tested in a multivariable model. The resulting hazard ratio (HR) was specified with the 95% confidence interval (CI), respectively.

Data administration and statistical analysis were conducted with the software Microsoft^®^ Excel (Microsoft Corp., Released 2024, Version 16., Redmond, WA, 2024 Microsoft), IBM^®^ SPSS Statistics for Windows (IBM Corp., Released 2023, Version 29.0.2.0, Armonk, NY: IBM Corp) and 'R' [R Core Team, R: A Language and Environment for Statistical Computing, R Foundation for Statistical Computing, version 4.0.2 plugin "KMWin" (28)]. A *p*-value less than 0.05 was considered statistically significant.

## 3 Results

### 3.1 Patient and disease characteristics

160 patients with ASCC treated with curative intent were included in this study. The median age at diagnosis was 62.6 years. 26.3% (n = 42) of the included patients were at least 70 years old and therefore constituted the "elderly group". All patients were treated by primary R(C)T. Median follow-up was 44.5 months (minimum: 2.0 months; maximum: 268.0 months). Patients' and disease characteristics of the entire study group as well as stratified by age group (≥ 70 years vs. < 70 years) are presented in **Table 1**. T-, N-categories and UICC-staging-categories as well as pathological grading and Body Mass Index (BMI) were relatively equally distributed. The CCI was significantly higher in the elderly group (**Table 1**; *p* < 0.01).

**Table 1:** Patients’, disease characteristics stratified by age group.

Characteristic	Total		Age group				*P*
			≥70 y		<70 y		
	[n]	[%]	[n]	[%]	[n]	[%]	
Total	160	100	42	100	118	100	
Age, median (min - max) (years)	62.6 (29.5 - 90.9)		77.3 (71.6 - 90.9)		58.0 (29.5 - 69.9)		**<0.01 ** ^a^
Sex							0.04 ^b^
Male	46	28.8	7	16,7	39	33.1	
Female	114	71.3	35	83.3	79	66.9	
BMI							0.36 ^c^
<25	64	40.0	14	33.3	50	42.4	
≥25	95	59.4	27	64.3	68	57.6	
No data	1	0.6	1	2.4	0	0.0	
Current/ former smoker							**<0.01** ^c^
yes	69	43.8	7	16.7	62	52.5	
no	64	40.0	28	66.7	36	30.5	
No data	27	16.3	7	16.7	20	16.9	
CCI							**<0.01 ** ^b^
1-3	55	34.4	1	2.4	54	45.8	
4-6	93	58.1	33	78.6	60	50.8	
≥7	12	7.5	8	19.0	4	3.4	
T category							0.59** ** ^b^
cT1	27	16.9	5	11.9	22	18.6	
cT2	64	40.0	20	47.6	44	37.3	
cT3	50	31.3	13	31.0	37	31.4	
cT4	19	11.9	4	9.5	15	12,7	
N category							0.23 ^b^
cN0	98	61.3	29	69.0	69	58.5	
cN1	62	38.8	13	31.0	49	41.5	
UICC classification							0.31** ** ^b^
I	21	13.1	5	11.9	16	13.6	
IIA	51	31.9	14	33.3	37	31.4	
IIB	18	11.3	8	19.0	10	8.5	
IIIA	22	13.8	7	16,7	15	12,7	
IIIB	9	5.6	2	4.8	7	5.9	
IIIC	39	24.4	6	14.3	33	28.0	
Grading							0.51 ^c^
G1	19	11.9	7	16,7	12	10.2	
G2	102	63.8	25	59.5	77	65.3	
G3	33	20.6	8	19.0	25	21.2	
No data	6	3.8	2	4.8	4	3.4	

^a^Kruskal-Wallis test.

^b^Pearson’s Chi-squared test.

^c^P-value of the Pearson’s Chi-squared test calculated without patients with no data.

BMI, body mass index; CCI, Charlson Comorbidity Index; min, minimum; max, maximum; UICC, Union internationale contre le cancer; y, years of age.

### 3.2 Treatment

Treatment details of the entire study group as well as stratified by age group are depicted in **Table 2**. There was no significant difference concerning RT administration, overall treatment or RT delivery techniques. The median planned and administered RT dose was 50.4 Gy in both groups, respectively. Neither the distribution of the RT technique nor the radiation dosage or completion rate revealed significant discrepancies between the groups (**Table 2**).

**Table 2:** Treatment details stratified by age group.

Characteristic	Total		Age group				*P*
			≥70 y		<70 y		
	[n]	[%]	[n]	[%]	[n]	[%]	
Total	160	100	42	100	118	100	
Radiotherapy							
Overall treatment time (median (min - max)) [d]	39 (27 - 74)		40 (31 - 74)		39 (27 - 72)		0.24 ^a^
Unscheduled radiotherapy treatment break							0.51** ** ^b^
No	113	70.6	28	66.7	85	72.0	
Yes	47	29.4	14	33.3	33	28.0	
Dosage (median (min - max))							
Prescribed [Gy]	50.4 (45.0 - 61.0)		50.4 (50.0 - 59.4)		50.4 (45.0 - 61.0)		0.58 ^a^
Achieved [Gy]	50.4 (45.0-61.0)		50.4 (43.2 - 59.4)		50.4 (36.0 - 61.0)		0.35 ^a^
Achieved of prescribed radiation dose							0.25** ** ^b^
100 %	141	88.1	35	83.3	106	89.8	
< 100 %	19	11.9	7	16,7	12	10.2	
Technique							0.06** ** ^b^
Conventional (2D/3D)	87	54.4	28	66,7	59	50.0	
Dynamic (IMRT/VMAT)	73	45.6	14	33.3	59	50.0	
Concomitant chemotherapy							**< 0.001 ** ^b^
No	9	5.6	7	16,7	2	1,7	
Yes	151	94.4	35	83.3	116	98.3	
Completion							0.41** ** ^b^
Incomplete	14	8.8	2	4.8	12	10.2	
Complete	137	85.6	33	78.6	104	88.1	
Regimen							0.43** ** ^b^
5-FU + Mitomycin C	144	90.0	34	81.0	110	93.2	
5-FU + Cisplatin	4	2.5	0	0.0	4	3.4	
Other	3	1.9	1	2.4	2	1,7	
Absolute dose applied [mg]							
5-FU	14200 (6000 - 18000)		13760 (7200 - 16000)		14360 (6000 - 18000)		0.18 ^a^
Mitomycin C	35.2 (10 - 41)		34.0 (18 - 38)		35.5 (10 - 41)		0.22 ^a^
Cisplatin	301.1 (131 - 400)		400.0 (400 - 400; n = 1)		255.8 (131 - 393)		**< 0.01** ^a^
Prior incomplete surgery							0.29** ** ^b^
No	151	94.4	41	97.6	110	93.2	
Yes	9	5.6	1	2.4	8	6.8	

^a^Kruskal-Wallis test.

^b^Pearson’s Chi-squared test.

d, days; Gy, Gray; IMRT, Intensity-modulated radiation therapy; mg, milligrams; min, minimum; max, maximum; VMAT, Volumetric modulated arc therapy; 5-FU, 5-fluorouracil; y, years of age.

Concomitant CTx was given in 94.4% (n = 151) of the entire study group. Elderly patients significantly less frequently received concomitant CTx (**Table 2**). In case of CTx application, the CTx regimens used as well as the completion rate were not significantly different distributed between elderly vs. younger patients (**Table 2**). Most patients received a 5-FU based CTx regimen (n = 148; 92.5%) and in most cases 5-FU was combined with mitomycin (n = 144 of 148; 97.5%). In case of poor lung function cisplatin instead of mitomycin was used (n = 4 of 148; 2.5%). The applied CTx absolute dosage showed no significant deviation between the groups except for Cisplatin, which was used only for one patient in the elderly group (**Table 2**).

### 3.3 Toxicity

Detailed results of the toxicity analyses are provided in **Table 3**. Acute toxicities were relatively equally distributed between elderly vs. younger patients, except for enteritis. Enteritis ≥ III° occurred more frequently in elderly patients. Regarding late toxicities, gastrointestinal/urinary toxicities showed no significant discrepancy between elderly vs. younger patients. Elderly compared to younger patients developed significantly more frequently bone fractures. Concerning elderly females, a significant lower rate of vaginal toxicity was reported. Among patients with CTx in the therapeutic concept the rates of acute toxicity ≥ III° showed no significant discrepancy between elderly and younger patients (45.7% (n= 16) vs. 37.9% (n=44), *p* = 0.41).

**Table 3:** Treatment related toxicities stratified by age group.

Characteristic	Total		Age group				*P*
			≥70 y		<70 y		
	[n]	[%]	[n]	[%]	[n]	[%]	
Total	160	100	42	100	118	100	
Acute organ toxicity							
≥III°	66	41.3			47	39.8	0.54 ^a^
Dermatitis							
no	3	1.9	2	4.8	1	0.8	0.44 ^a^
I°	17	10.6	4	9.5	13	11.0	
II°	88	55.0	22	52.4	66	55.9	
III°	52	32.5	14	33.3	38	32.2	
IV°	0	0.0	0	0.0	0	0.0	
≥III°	52	32.5	14	33.3	38	32.2	0.89 ^a^
Enteritis							
no	58	36.3	15	35.7	43	36.4	0.09 ^a^
I°	62	38.8	14	33.3	48	40,7	
II°	30	18.8	7	16,7	23	19.5	
III°	0	0.0	0	0.0	0	0.0	
IV°	10	6.3	6	14.3	4	3.4	
≥III°	10	6.3	6	14.3	4	3.4	0.01 ^a^
Procitits							
no	35	21.9	11	26.2	24	20.3	0.14 ^a^
I°	64	40.0	12	28.6	52	44.1	
II°	56	35.0	16	38.1	40	33.9	
III°	5	3.1	3	7.1	2	1,7	
IV°	0	0.0	0	0.0	0	0.0	
≥III°	5	3.1	3	7.1	2	1,7	0.08 ^a^
Cystitis							
no	89	55.6	18	42.9	71	60.2	0.01 ^a^
I°	50	31.3	15	35,7	35	29,7	
II°	16	10.0	9	21.4	7	5.9	
III°	5	3.1	0	0.0	5	4.2	
IV°	0	0.0	0	0.0	0	0.0	
≥III°	5	3.1	0	0.0	5	4.2	0.18 ^a^
Hematologic							
≥III°	38	23.8	9	21.4	29	24.6	0.68 ^a^
Anemia							
no	80	50.0	18	42.9	62	52.5	0.51 ^a^
I°	50	31.3	13	31.0	37	31.4	
II°	25	15.6	9	21.4	16	13.6	
III°	5	3.1	2	4.8	3	2.5	
IV°	0	0.0	0	0.0	0	0.0	
≥III°	5	3.1	2	4.8	3	2.5	0.48 ^a^
Leukopenia							
no	40	25.0	13	31.0	27	22.9	0.24 ^a^
I°	37	23.1	11	26.2	26	22.0	
II°	55	34.4	9	21.4	46	39.0	
III°	26	16.3	9	21.4	17	14.4	
IV°	2	1.3	0	0.0	2	1,7	
≥III°	28	17.5	9	21.4	19	16.1	0.43 ^a^
Thrombopenia							
no	107	66.9	32	76.2	v	76.2	0.50 ^a^
I°	30	18.8	7	16,7	7	16,7	
II°	9	5.6	1	2.4	1	2.4	
III°	11	6.9	2	4.8	2	4.8	
IV°	3	1.9	0	0.0	0	0.0	
≥III°	14	8.8	2	4.8	2	4.8	0.29 ^a^
Late toxicity							
≥II°	29	18.1	10	23.8	19	16.4	0.29 ^a, b^
No data	2	1.3	0	0	2	1.7	
Gastrointestinal/ urinary							
I-IV°	17	10.6	5	11.9	12	10.2	0.76 ^a, b^
No data	2	0.1	0	0	2	0,1	
Vaginal (among females)							
I-IV°	14	8.8	1	2.4	13	11.0	**0.05** ^a, b^
No data	10	6.2	4	11.4	6	5,1	
Pelvic bone fracture							
I-IV°	7	4.4	5	11.9	2	1,7	**<0.01** ^a, b^
No data	10	6.2	5	11.9	5	4.2	

^a^Pearson’s Chi-squared test.

^b^Patients without data were excluded from the respective analysis.

y, years of age.

### 3.4 Survival

**Figure 1:**
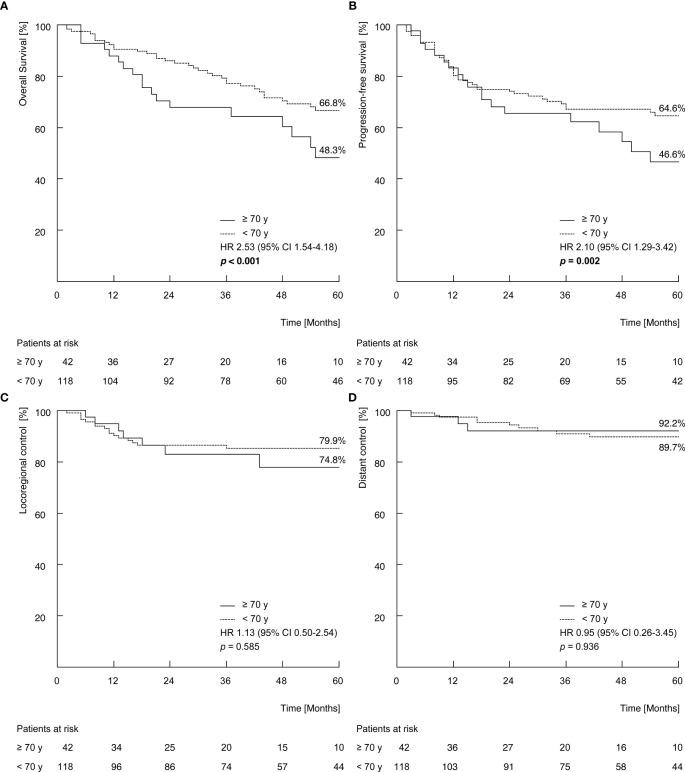
Five-year Kaplan-Meier estimates of overall survival **(A)**, disease-specific survival **(B)**, locoregional control **(C)** and distant control **(D)** stratified by age group (≥70 years vs. <70 years). *P*-values calculated by log-rank test. y = age of life in years.

In the entire study group, the five-year estimates for OS, PFS, LRC and DC were 62.0%, 60.2%, 78.9%, 90.1%, respectively. Elderly patients showed a significant inferior OS (48.3% vs. 66.8%, *p* < 0.001; **Figure 1A**) and PFS (46.6% vs. 64.6%, *p* = 0.002; **Figure 1B**). No significant difference between elderly and younger patients was observed for LRC (74.8% vs. 79.9%, p = 0.775; **Figure 1C**) and DC (92.2% vs. 89.7%, *p* = 0.926; **Figure 1D**).

To identify potential prognostic relevant confounders, the association of baseline, treatment and toxicity characteristics and survival were analyzed in a univariable cox-regression analysis, respectively. Therefore, patients were stratified according to characteristics, that were also analyzed for age group dependent discrepancies (**Tables 1**-**3**). Results are demonstrated in **Table 4**. In the univariable analysis characteristics associated with OS as well as PFS were age ≥ 70 years, UICC stage III as well as application of concomitant CTx, a CCI ≥ 5 and development of an acute toxicity ≥ III°, respectively. Additionally, PFS was also associated with the development of a late toxicity ≥ III°. Characteristics associated with LRC in the univariable analysis were concomitant CTx and development of an acute development of an acute toxicity ≥ III°, respectively. Regarding DC, a significant result was reported for the development of acute and late toxicity ≥ III°, respectively.

**Table 4:** Univariate analysis of baseline, treatment and toxicity characteristics and survival.

Characteristic	OS		PFS		LRC		DC		SFS	
	HR (95% CI)	*P* ^a^	HR (95% CI)	*P* ^a^	HR (95% CI)	*P* ^a^	HR (95% CI)	*P* ^a^	HR (95% CI)	*P* ^a^
**Age**										
per year	1.04 (1.02-1.06)	**<0.001**	1.04 (1.01-1.06)	**0.002**	1.02 (0.99-1.05)	0.26	0.99 (0.95-1.05)	0.95	1.02 (0.99-1.05)	0.06
≥70 y (n=42) vs. <70 y (n=118)	2.53 (1.54-4.18)	**<0.001**	2.10 (1.29-3.42)	**0.003**	1.13 (0.50-2.54)	0.78	0.95 (0.26-3.45)	0.99	1.85 (1.07-3.20)	**0.03**
**Current/ former smoker**										
Yes (n=69) vs. no (n=64)	1.50 (0.89-2.53)	0.13	1.52 (0.91-2.53)	0.11	1.17 (0.52-2.61)	0.71	1.71 (0.50-5.83)	0.39	1.28 (0.72-2.27)	0.40
**BMI** ^b^										
≥25 (n=95) vs. <25 (n=64)	0.67 (0.42-1.07)	0.09	0.64 (0.41-1.01)	0.06	0.87 (0.42-1.83)	0.72	1.46 (0.45-4.74)	0.53	0.93 (0.55-1.56)	0.78
**T category**										
cT4 (n=19) vs. cT1-3 (n=141)	1.45 (0.69-3.05)	0.32	1.72 (0.88-3.37)	0.11	1.50 (0.52-4.32)	0.45	0.76 (0.99-5.83)	0.79	2.13 (0.76-5.98)	0.15
**UICC classification**										
III (n=70) vs. ≤ II (n=90)	2.53 (1.54-4.18)	**<0.001**	2.10 (1.29-3.42)	**0.003**	1.28 (0.53-3.11)	0.58	0.95 (0.26-3.45)	0.94	1.95 (1.07-3.20)	**0.03**
**N category**										
N+ (n=62) vs. N0 (n=98)	1.42 (0.88-2.28)	0.15	1.41 (0.88-2.24)	0.15	1.72 (0.83-3.57)	0.15	2.07 (0.69-6.15)	0.19	1.74 (1.01-3.02)	**0.05**
**Grading**										
G3 (n=33) vs. G1-2 (n=121)	0.83 (0.45-1.52)	0.55	0.84 (0.47-1.50)	0.55	0.97 (0.39-2.38)	0.94	0.31 (0.04-2.39)	0.26	0.94 (0.49-1.79)	0.85
**Radiotherapy**										
Incomplete (n=20) vs. complete (n=140)	1.03 (0.53-1.98)	0.94	1.24 (0.67-2.28)	0.50	1.36 (0.47-3.90)	0.57	1.51 (0.33-6.80)	0.59	1.45 (0.74-2.81)	0.28
Technique, dynamic (n=73) vs. conventional (n=87)	0.90 (0.53-1.53)	0.69	0.70 (0.42-1.18)	0.18	0.85 (0.40-1.78)	0.66	0.53 (0.16-1.72)	0.29	0.83 (0.44-1.56)	0.57
**Concomitant chemotherapy**										
Yes (n=151) vs. no (n=9)	0.24 (0.12-0.49)	**<0.001**	0.27 (0.15-0.55)	**<0.001**	0.18 (0.07-0.47)	**<0.001**	0.23 (0.05-1.04)	0.06	0.19 (0.07-0.48)	**<0.01**
incomplete (n=14) vs. complete (n=137)	1.34 (0.64-2.83)	0.44	1.33 (0.63-2.80)	0.45	1.30 (0.39-4.37)	0.67	3.33 (0.89-12.57)	0.08	0.84 (0.36-1.98)	0.69
**CCI**										
≥5 (n=66) vs. <5 (n=94)	2.57 (1.60-4.14)	**<0.001**	2.37 (1.47-3.81)	**<0.001**	1.27 (0.61-2.64)	0.53	1.40 (0.47-4.19)	0.45	2.44 (1.43-4.17)	**<0.01**
**Acute toxicity ≥ III°**										
Yes (n=62) vs. no (n=98)	0.35 (0.21-0.59)	**<0.001**	0.39 (0.17-0.52)	**<0.001**	0.09 (0.21-0.38)	**0.001**	0.21 (0.04-0.97)	**0.04**	0.37 (0.20-0.69)	**0.001**
**Late toxicity ≥ III°** ^b^										
Yes (n=17) vs. no (n=141)	1.84 (0.94-3.62)	0.08	2.15 (1.12-4.10)	**0.02**	1.57 (0.55-4.50)	0.41	9.26 (3.10-27.61)	**<0.001**	1.71 (1.03-2.85)	**0.04**

P-value of Cox regression analysis.

Patients without data were excluded from the respective analysis.

BMI, body mass index; CCI, Charlson Comorbidity Index; CI, confidence interval; DC, distant control; HR, hazard ratio; LRC, locoregional control; min, minimum; max, maximum; OS, overall survival; PFS, progression-free survival; UICC, Union internationale contre le cancer; SFS, stoma-free survival; y, years of age.

Detailed results of the multivariable analyses are shown in **Table 5**. Multivariable analyses revealed both, OS and PFS, were significantly influenced by patients' age, the application of concomitant CTx and/or the development of acute toxicity ≥ III°, respectively. Furthermore, multivariable analyses showed, LRC was significantly influenced by the application of CTx and/or the development of acute toxicity ≥ III°, and DC by the development of acute toxicity ≥ III° and/or late toxicity ≥ III°, respectively.

**Table 5:** Multivariable analysis of baseline, treatment and toxicity characteristics and survival.

Characteristic ^a^	OS		PFS		LRC		DC		SFS	
	HR (95% CI)	*P* ^b^	HR (95% CI)	*P* ^b^	HR (95% CI)	*P* ^b^	HR (95% CI)	*P* ^b^	HR (95% CI)	*P* ^b^
**Age**										
≥70 y (n=42) vs. <70 y (n=118)	2.43 (1.38-4.27)	**0.002**	2.25 (1.29-3.89)	**0.004**	/		/		1.16 (0.51-2.97)	0.72
**UICC classification**										
III (n=70) vs. ≤ II (n=90)	1.38 (0.85-2.25)	0.18	1.56 (0.97-2.51)	0.06	/		/		1.85 (1.04-3.30)	**0.036**
**Concomitant chemotherapy**										
Yes (n=151) vs. no (n=9)	0.40 (0.18-0.88)	**0.023**	0.40 (0.19-0.86)	**0.02**	0.24 (0.08-0.71)	**0.01**	/		0.21 (0.08-0.59)	**0.002**
**CCI**										
≥5 (n=66) vs. <5 (n=94)	1.27 (0.63-2.54)	0.51	1.36 (0.67-2.73)	0.39	/		/		2.56 (1.46-4.46)	**<0.001**
**Acute toxicity ≥ III°**										
Yes (n=62) vs. no (n=98)	0.31 (0.18-0.53)	**<0.001**	0.28 (0.16-0.49)	**<0.001**	0.12 (0.03-0.51)	**0.004**	0.24 (0.05-1.09)	**0.06**	0.32 (0.17-0.61)	**<0.001**
**Late toxicity ≥ III°**										
Yes (n=17) vs. no (n=141)	/		1.59 (0.79-3.16)	0.19	/		8.47 (2.83-25.34)	**<0.001**	3.12 (1.19-8.21)	**0.021**

Descriptive data refers to the entire study group

P-value of Cox regression analysis.

CCI, Charlson Comorbidity Index; CI, confidence interval; DC, distant control; HR, hazard ratio; LRC, locoregional control; OS, overall survival; PFS, progression-free survival; SFS, stoma-free survival; y, years of age.

### 3.5 Stoma therapy

**Figure 2:**
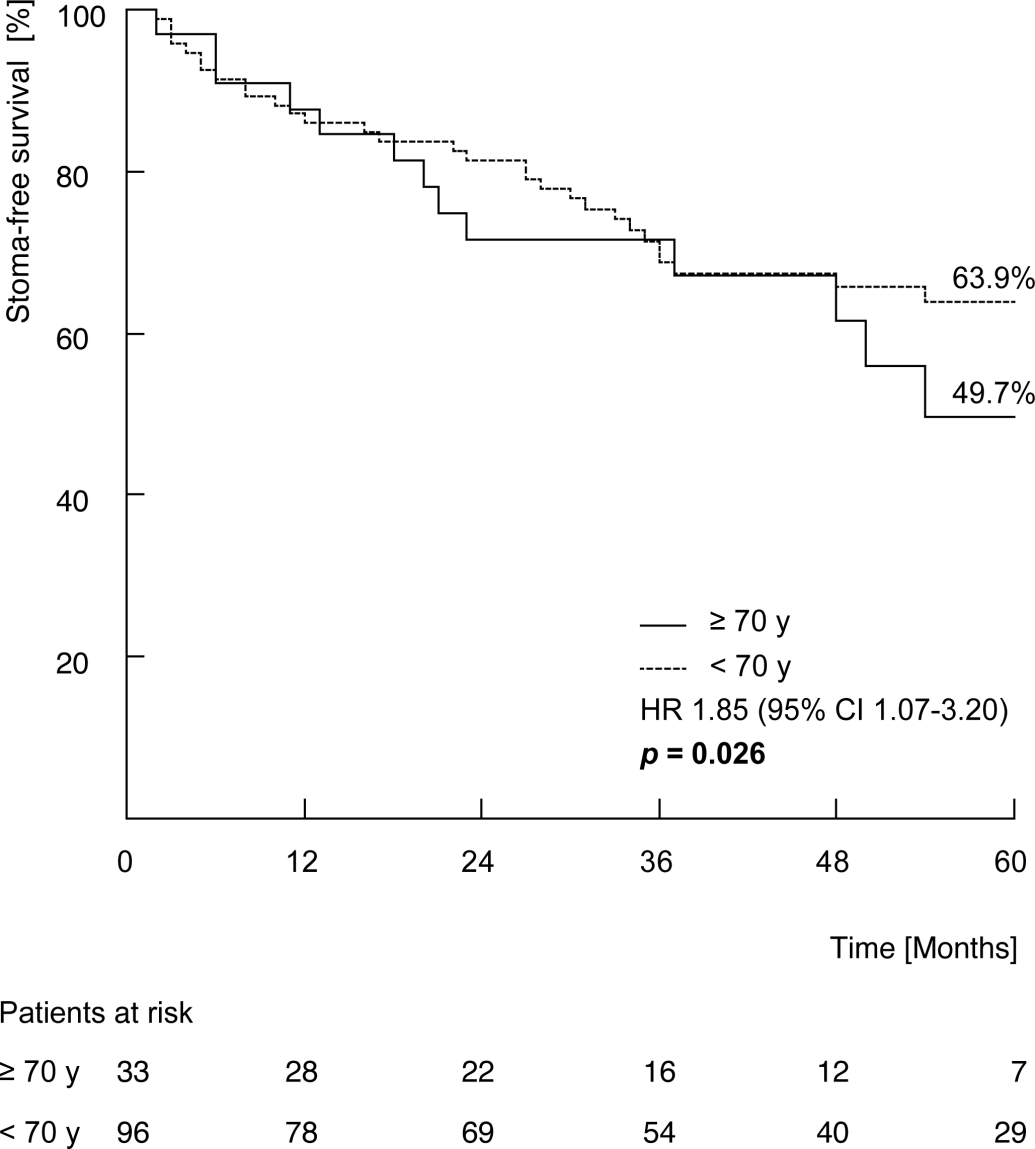
Five-year Kaplan-Meier estimates of stoma-free survival stratified by age group (≥70 years vs. <70 years). *P*-values calculated by log-rank test. y = age of life in years.

Regarding stoma therapy, there was no significant difference in frequency of prophylactic or posttherapeutic colostomy or colostomies performed during the therapy period between elderlies and younger patients (prophylactic: 9 (21.4%), vs. 22 (18.6%) *p* = 0.695; posttherapeutic: 4 (12.1%) vs. 12 (12.5%), *p* = 0.955; during therapy: 0 (0%) vs. 2 (1.7%)). Among patients without a prophylactic colostomy, elderly patients showed a significant lower SFS (49.7% vs. 63.9%, *p* = 0.026; **Figure 2**). Furthermore, univariable analysis showed a significant association between a superior SFS and concomitant CTx and the occurrence of acute toxicity ≥ III°, respectively (**Table 4**). Worse SFS was associated with age ≥ 70 years, nodal involvement, UICC stage III, CCI ≥ 5 and the development of late toxicity ≥ III°, respectively (**Table 4**). In the multivariable analyses UICC stage III, the application of concomitant CTx, a CCI ≥ 5 as well as the development of acute toxicity ≥ III° and late toxicity ≥ III° remained significant variables influencing SFS, respectively (**Table 5**).

### 3.6 Subgroup analyses

To address the advances of radiation techniques during the inclusion period, the subgroup of the 14 elderly and 59 younger patients treated with VMAT/IMRT was analyzed separately. Results of the univariate cox regression for this subgroup are provided in **Supplementary Table 1**. There were no significant prognostic differences between older compared to younger patients. The respective log-rank test of the survival endpoints also revealed no significant differences between older compared to younger patients (OS: *p* = 0.254; PFS: *p* = 0.373; LRC: *p* = 0.476; DC: p = 0.694; SFS: *p* = 0.816).

Toxicity analyses revealed, that there was neither a significant difference in rate of acute toxicities ≥ III° (n = 3 vs. 12; *p* = 0.928) nor late toxicities ≥ III° (n = 1 vs. 5, *p* = 0.844) between elderly compared to younger patients.

## 4 Discussion

This study presents a retrospective analysis of patients with non-metastasized anal cancer treated with primary R(C)T with curative intent. The focus was on elderly patients, who have a higher prevalence of multiple chronic diseases (29) and therefore potential competing risk factors for an adverse outcome (30). Elderly and/or comorbid patients are frequently underrepresented or even excluded from pivotal studies (6–12). Consequently, it is questionable whether these studies' results are transferable in case of advanced age and/or presence of comorbidities. The highest evidence for the treatment of elderly patients relies on a small number of retrospective studies. To the best of our knowledge only four of them reported five-year oncological outcome data (13–16). Despite relatively long inclusion periods in all of them, the studies included only a relatively small number of patients (N = 76 - 278) (13–16). The most likely reason is the rarity of anal carcinomas (31). Information concerning patients' CCI is only given by one study (13). However, this study did not include a younger comparison group and thus did not enable a comparison of the influence of comorbidities on therapeutic outcomes between elderly and younger patients. Comparability of published studies may be further reduced by the heterogeneous treatment concepts reported (13, 14, 16).

Another difficulty is the variety of the definition of "elderly" patients in literature. In the present study the age cut-off was chosen in line with other studies focusing on elderly anal cancer patients (13, 14, 16, 32) and to be approximately ten years above the median age of the complete study group, which was considered a sufficient method to study especially old/very old patients (33).

In the present study, descriptive analyses revealed elderly patients had less frequently a positive smoking history. This is in line with the higher risk of anal cancer development in advanced age (2, 34) as well as the higher risk of anal cancer development in younger patients with a positive smoking status (35). The higher proportion of elderly patients with a higher CCI indicate that geriatric oncological care more often involves also the management of accompanying comorbidities. In line with this, despite comparable tumor characteristics a larger proportion of elderly compared to younger patients received RT without concomitant CTx. The less frequent use of CTx in the oncogeriatric setting was also described by others (14, 15). Potential reasons could be the higher rate of contradictions for CTx (e.g. cardiac and/or renal dysfunction) or even a reduced general condition and the reported increased risk of side effects associated with CTx in elderly patients (36, 37). Nevertheless, in order to enable as many people as possible to benefit from the expected prognostic advantage of concomitant CTx (11, 12), in case of contraindications to the standard regimen adaptation of dose and schedule to the specific clinical condition is an accepted procedure (4, 14, 38, 39). Despite the adapted treatment regimens in case of contradictions to the standard, the cumulative dose of the respectively used chemotherapeutic agents did not show any significant discrepancies between elderly vs. younger patients in the present study. Alike there was no significant difference in the main parameters of RT administration. This indicates a feasibility of the respective treatment regimens and indicates an adequate patient selection process. It supports the recommendation of the European Society for Medical Oncology (ESMO) to treat elderly patients similarly to younger ones (40).

A good treatment efficiency for elderly and younger patients was indicated by comparable high disease-related outcomes (LRC, DC). This is in line with previous studies describing no significant difference between the estimates for LCR (16), DFS and DC (15, 16). Including also patients who were treated with palliative doses due to a "poor performance status and/or comorbidities", Claren et al. describes a consequently significant reduced DC, LCR, DFS in elderly patients (14). The present study as well as the others mentioned above (15, 16) support the ESMO recommendation to treat elderly patients similarly to younger ones (40) and RCT as a potential curative therapy also in in elderly and comorbid patients. The prognostic relevance of concomitant CTx is strengthened by the fact that multivariate analysis revealed it as an independent prognostic factor of a significant superior OS, PFS as well as LRC also in the present study. Nevertheless, the present study also highlights the importance of RT as the backbone of treatment, since the disease-related outcomes were, as described above, comparably high despite a lower proportion of elderly patients with CTx in the treatment concept.

The lower rate of chemotherapeutic treatment in elderly patients may also be a reason for the shorter SFS in elderly patients without pretherapeutic colostomy. In line with this Bartelink et al. reported a 32% longer colostomy-free survival in patients treated with RCT compared to those treated with RT (11). Furthermore, the present study's multivariable analyses revealed a positive association between a superior SFS and concomitant CTx. A negative prognostic association was identified between a CCI ≥ 5, UICC stage III and SFS. The results of the present study therefore underline the necessity to inform patients prior to therapy, that even if organ preservation is possible, the rate of failure may be higher in patients with contradictions to CTx, a CCI ≥ 5 and a more advanced stage disease (UICC stage III).

Regarding OS and PFS, it needs to be discussed that despite the good disease-related outcomes indicated a comparable treatment efficiency in younger and elderly patients, the present studies' elderly patients showed significantly lower estimates compared to younger ones. This was also confirmed in the multivariable analyses. In line with these results Dale et al. reported similar CR, local and distant failures and DFS, but a significant lower CSS for elderly patients (15). In contrast two other studies reported no significant difference in OS between elderly versus younger patients treated with curative intent (14, 16). Unfortunately, none of these studies reports on comorbidities within the respective groups (14–16). The results of the present study are most likely linked to the age-related reduced life expectancy and to the higher prevalence of comorbidities in elderlies. In the present study 97.6% of elderly compared to 54.2% of younger patients had CCI ≥ 4, corresponding to a 1-year mortality of ≥ 52% (41). Moreover, 19.0% of elderly compared to 3.4% of younger patients had a CCI ≥ 7, corresponding to a 10-year survival of 0% (41). The relevance of taking competing risk factors into account is also supported by the results of the ACT-I trial. Referring to the 13-year follow-up data of the ACT-I trial, at least approximately 20% of patients died due to others causes of e.g. cardiac origin (6), although patients considered too old or too unfit were excluded from randomization (7). Taken together, this once again underlines the need for adequate patient selection, education and care.

Evaluation of acute toxicities revealed comparable rates of cutaneous and hematological toxicity ≥ III° in the present study. The higher frequency of enteritis ≥ III° in elderly patients indicated the higher vulnerability of this group for radiation induced gastrointestinal changes and the importance and relevance of close and continuous surveillance and sufficient supportive care. Possible and interdependent causes for a higher rate of acute enteritis in elderly patients could be the higher rate of preexisting comorbidities, potentially associated also with a higher frequency of (poly)medication with impact on gastrointestinal function and age-related changes in the organ function itself (42). In line with this in the present study was no significant difference between the rate of gastrointestinal/urinary toxicity three months after therapy between older versus younger patients observed. Furthermore, in the present study, the occurrence of pronounced early toxicity proved to be prognostically favorable in terms of OS, PFS and LRC. This phenomenon has already been observed in previous studies (43) and could, for example, be an expression of increased radiosensitivity of tumor and normal tissue.

Concerning late toxicities, elderly females had a significant lower rate of vaginal toxicity. This may most likely be due to a lower sexual activity as well as pre-existing discrepancies and potentially also a reporting bias. A potential explanation for the more frequent pelvic fractures in elderly patients could be the higher probability of pre-existing osteoporosis or corresponding risk factors (e.g. vitamin D deficiency, inactivity) in elderly patients (44, 45). This should again sensitize to screen especially elderly patients for presence of risk factors for osteoporosis and, if possible, to counteract it therapeutically or preventively. Potential approaches to reduce pelvic fractures associated with pelvic RT could be the reduction of risk factors of osteoporosis, multicausal bone loss by e. g. bisphosphonates (46) or even risk-adapted reduction of radiation dose to pelvic bone substructures related to an increased risk of pelvic insufficiency fractures (47). Furthermore, in the present study, the occurrence of late toxicity ≥ III° was identified as a negative prognostic factor for poorer DC in the multivariable analyses. This result cautions for closer follow-up and early search for distant tumor manifestations, especially in cases of higher-grade late toxicity.

The relevance of the present study is emphasized by the lack of detailed high-evidence data concerning R(C)T for elderly and comorbid patients with anal cancer. Only a paucity of only retrospective studies addressed this topic with a focus on long-term oncological outcome of patients with anal cancer treated with curative intent (13–16). Like the current study, all of them have relatively long inclusion periods. Therefore, the progress made in oncological management probably cannot be taken into account sufficiently [e.g. establishment of VMAT (17)]. Consequently, also reported survival data might be under- and toxicity might be overestimated with regard to current standards. Although the present study also presents results separately for the group of patients treated with VMAT/IMRT, these results should be interpreted with caution, particularly due to the small number of patients included. To address the limited data quality and considerable risk of bias to retrospective studies, detailed clinicopathologic data from original patient charts, surgical and pathologic reports were provided. This allowed to analyze the prognostic impact of patient, disease and treatment characteristics in a well-characterized group of patients with anal cancer treated in a radiotherapeutic concept with curative intent. To the best of our knowledge, it is the only study, that focused on the relevance of advanced compared to younger age and included, with the CCI, also more comprehensive analyses of comorbidities. The present study provides evidence that will potentially influence the inclusion criteria and analytic scope of future trials, focusing on elderly patients. Therapeutic strategies should be developed in interdisciplinary conferences and investigated in prospective randomized trials with advanced patient involvement, quality of life and socioeconomic analyses.

## 5 Conclusion

In the clinical routine of a University Medical Center, most elderly patients received the standard treatment of R(C)T. The comparable high disease related outcomes combined with the high therapy completion rate as well as its tolerability indicate the feasibility, safety and effectiveness of R(C)T also in elderly patients with anal cancer. A sufficient patient selection process including also a prior geriatric assessment and a comprehensive pretherapeutic discussion of potential benefits and treatment associated side effects is essential for optimized patient-centered and personalized care. An adequate supportive therapy concept and follow-up regimen should also be of particular importance. Especially an advanced age at diagnosis as well as a CCI ≥ 5 are pre-therapeutic characteristics, which could negatively influence oncological results. The development of higher-grade late toxicity should caution for a closer follow-up and early search for distant tumor manifestations.

## Data Availability

The original contributions presented in the study are included in the article/**Supplementary Material**. Further inquiries can be directed to the corresponding author.
