# Supplementary material for: Radio(chemo)therapy with curative intent for anal cancer – effectiveness and toxicity in elderly vs. younger patients
Source: Front Oncol. 2025 May 16;15:1567655. doi: 10.3389/fonc.2025.1567655 (PMC12122431; doi:10.3389/fonc.2025.1567655)
Supplement: Supplementary file 1 [file Table1.docx]

|  | | | | | | | | | | | |
| --- | --- | --- | --- | --- | --- | --- | --- | --- | --- | --- | --- |
| **TABLE 5.**  Univariate analysis of baseline, treatment and toxicity characteristics and survival. | | | | | | | | | | | |
|  | | | | | | | | | | | |
| **Characteristic** | | **OS** | | **PFS** | | **LRC** | | **DC** | | **SFS** | |
|  |  | **HR (95% CI)** | ***P* ^a^** | **HR (95% CI)** | ***P* ^a^** | **HR (95% CI)** | ***P* ^a^** | **HR (95% CI)** | ***P* ^a^** | **HR (95% CI)** | ***P* ^a^** |
| **Age** | |  |  |  |  |  |  |  |  |  |  |
|  | per year | 1.04 (0.99-1.08) | 0.06 | 1.04 (0.99-1.08) | 0.07 | 1.04 (0.99-1.09) | 0.12 | 1.06 (0.97-1.16) | 0.19 | 0.99 (0.95-1.04) | 0.06 |
|  | ≥70 y (n=14) vs. <70 y (n=59) | 1.73 (0.67-4.50) | 0.26 | 1.54 (0.59-3.98) | 0.38 | 1.60 (0.43-5.93) | 0.48 | 1.59 (0.16-15.09) | 0.69 | 0.84 (0.19-3.72) | 0.82 |
| **Current/ former smoker** | |  |  |  |  |  |  |  |  |  |  |
|  | Yes (n=40) vs. no (n=26) | 1.97 (0.72-5.43) | 0.19 | 2.70 (0.89-8.15) | 0.08 | 3.54 (0.76-16.39) | 0.11 | 1.30 (0.12-14.36) | 0.93 | 2.83 (0.79-10.19) | 0.40 |
| **BMI** ^b^ | |  |  |  |  |  |  |  |  |  |  |
|  | ≥25 (n=95) vs. <25 (n=64) | 0.48 (0.21-1.10) | 0.09 | 0.45 (0.20-1.05) | 0.06 | 0.64 (0.20-2.00) | 0.44 | 1.55 (0.19-14.96) | 0.70 | 1.45 (0.49-4.29) | 0.50 |
| **T category** | |  |  |  |  |  |  |  |  |  |  |
|  | cT4 (n=12) vs. cT1-3 (n=61) | 1.64 (0.55-4.89) | 0.32 | 2.12 (0.76-5.91) | 0.14 | 2.37 (0.64-8.75) | 0.19 | 0.04 (0.00-19755) | 0.63 | 1.44 (0.32-6.43) | 0.64 |
| **UICC classification** | |  |  |  |  |  |  |  |  |  |  |
|  | III (n=40) vs. ≤ II (n=33) | 0.99 (0.42-2.36) | 0.99 | 1.18 (0.47-2.64) | 0.80 | 1.44 (0.45-4.54) | 0.58 | 0.89 (0.13-6.32) | 0.91 | 1.11 (0.39-3.08) | 0.85 |
| **N category** | |  |  |  |  |  |  |  |  |  |  |
|  | N+ (n=35) vs. N0 (n=38) | 0.80 (0.33-1.84) | 0.62 | 0.93 (0.39-2.21) | 0.87 | 1.72 (0.83-3.57) | 0.15 | 1.06 (0.15-7.53) | 0.95 | 1.14 (0.41-3.18) | 0.80 |
| **Grading** | |  |  |  |  |  |  |  |  |  |  |
|  | G3 (n=21) vs. G1-2 (n=47) | 0.59 (0.22-1.65) | 0.32 | 0.88 (0.34-2.29) | 0.79 | 1.25 (0.40-3.90) | 0.70 | 0.02 (0.00-455) | 0.46 | 0.78 (0.23-2.59) | 0.69 |
| **Radiotherapy** | |  |  |  |  |  |  |  |  |  |  |
|  | Incomplete (n=6) vs. complete (n=67) | 5.92 (1.94-18.05) | **0.002** | 6.99 (2.52-19.42) | **<0.001** | 2.19 (0.28-17.11) | 0.42 | 0.05 (0.00-8x10^4) | 0.78 | 2.35 (0.53-10.47) | 0.26 |
| **Concomitant chemotherapy** | |  |  |  |  |  |  |  |  |  |  |
|  | Yes (n=70) vs. no (n=3) | 0.48 (0.13-1.80) | 0.29 | 0.40 (0.11-1.49) | 0.17 | 0.15 (0.03-0.67) | **0.013** | 0.12 (0.01-1.19) | 0.07 | n.a. |  |
|  | incomplete (n=8) vs. complete (n=62) | 1.83 (0.61-5.53) | 0.29 | 2.23 (0.74-6.74) | 0.16 | 3.72 (0.96-14.42) | 0.06 | 13.76 (1.25-151.99) | **0.030** | 0.44 (0.06-3.37) | 0.43 |
| **CCI** | |  |  |  |  |  |  |  |  |  |  |
|  | ≥5 (n=30) vs. <5 (n=43) | 2.26 (0.97-5.28) | 0.06 | 1.93 (0.83-4.51) | 0.13 | 1.69 (0.54-5.26) | 0.37 | 4.95 (0.52-47.77) | 0.16 | 2.22 (0.79-6.19) | 0.13 |
| **Acute toxicity ≥ III°** | |  |  |  |  |  |  |  |  |  |  |
|  | Yes (n=15) vs. no (n=58) | 1.03 (0.37-2.92) | 0.96 | 0.49 (0.15-1.70) | 0.27 | 0.04 (0.01-14.17) | 0.27 | 0.03 (0.00-701.91) | 0.50 | 1.45 (0.46-4.61) | 0.52 |
| **Late toxicity ≥ III°** ^b^ | |  |  |  |  |  |  |  |  |  |  |
|  | Yes (n=6) vs. no (n=65) | 0.54 (0.94-4.05) | 0.55 | 0.52 (0.07-3.89) | 0.52 | 0.84 (0.11-6.54) | 0.87 | 3.25 (0.34-31.31) | 0.31 | 3.34 (0.91-12.18) | 0.07 |
| ^a^ P-value of Cox regression analysis.  ^b^ Patients without data were excluded from the respective analysis.  Abbreviations:  BMI, body mass index; CCI, Charlson Comorbidity Index; CI, confidence interval; DC, distant control; HR, hazard ratio; LRC, locoregional control; min, minimum; max, maximum; OS, overall survival; PFS, progression-free survival; UICC, Union internationale contre le cancer; SFS, stoma-free survival; y, years of age. | | | | | | | | | | | |
